# Evaluation of a novel deep tissue transvaginal near-infrared laser and applicator in an ovine model

**DOI:** 10.1007/s10103-021-03315-z

**Published:** 2021-04-14

**Authors:** Ralph Zipper, Brian Pryor

**Affiliations:** 1 UroShape, LLC, 200 S. Harbor City Blvd, Suite 401, Melbourne, FL 32901 USA; 2 Litecure, LLC, 101 Lukens Dr, STE A, New Castle, DE 19720 USA

**Keywords:** Photobiomodulation, Pelvis, Pain, Irradiance, Therapy

## Abstract

Photobiomodulation therapy (PBMT) is an effective means of treating muscle spasm and pain. A novel near-infrared laser system has been commercialized for the treatment of myofascial pelvic pain in women (SoLá Therapy, UroShape, LLC). This study was undertaken to determine if this device is capable of delivering therapeutic levels of irradiance to the pelvic muscles and to identify the surface irradiance required to achieve this goal. This novel class IV near-infrared laser and transvaginal applicator were used to deliver near-infrared light energy through the vaginal mucosa of an adult Suffolk/Dorset Ewe. Irradiance was measured on the surface of the levator ani muscle, inside the levator ani muscle, and inside the bladder. Measurements were taken at powers of 5 W and 0.5 W. 3.0% of vaginal surface irradiance was measured inside of the levator ani muscle. 4.4% of vaginal surface irradiance was measured inside the bladder. At 5 W, the novel laser system provided a surface irradiance of 738 mW/cm^2^. At 0.5 W, the system provided a surface irradiance of 74 mW/cm^2^. A novel class IV near-infrared laser and transvaginal applicator delivered therapeutic irradiance to the levator ani muscle and bladder of an anesthetized ewe at a power setting of 5 W. A power setting of 0.5 W failed to deliver therapeutic energy into either the levator ani muscle or bladder. Clinical applications targeting deeper tissues such as the pelvic muscles and or bladder should consider power settings that exceed 0.5 W and or irradiance of ≥ 75 mW/cm^2^.

## Introduction

Thousands of published laboratory studies and hundreds of published randomized controlled trials have described and defined the beneficial effects of near-infrared (NIR) light energy on living tissue, photobiomodulation [1]. The benefits of NIR energy have been demonstrated on conditions including sprain and strains, post-surgical pain, whiplash, muscular back pain, radiculopathy, tendinitis, and chronic conditions such as osteoarthritis rheumatoid arthritis, neck and back pain, epicondylitis, carpal tunnel syndrome, tendinopathy, fibromyalgia, plantar fasciitis, and chronic regional pain syndrome, as well as neuropathic pain conditions such as postherpetic neuralgia, trigeminal neuralgia, and diabetic neuropathy [1]. Cotler et al., in their review of the scientific and medical literature, including nearly 100 publications, noted that “The long-term effects of low-level laser therapy (NIR) occur within a week or two and can last for months and sometimes years as a result of improved tissue healing.” [1]. These authors also pointed out that there are four targets of NIR energy, trigger points to reduce tenderness and relax contracted muscle fibers, nerves to induce analgesia, the site of injury to promote healing, remodeling, and reduce inflammation, and lymph nodes to reduce edema and inflammation.

It is estimated that approximately 14% of adult women suffer from chronic pelvic pain (CPP) [2]. A recent study of almost 50,000 female US veterans found a 30% incidence of CPP and a 16.8% incidence of opioid use amongst sufferers [3]. The incidence of opioid use amongst CPP patients in the general population is consistent with the military cohort [4]. The majority of CPP sufferers share a treatable pathology, hypertonicity, and tenderness of the pelvic muscles, levator myalgia (myofascial pelvic pain). It is estimated that 60 to 85% of women with CPP have levator myalgia [4, 5]. Alleviation of this tender pelvic muscle hypertonicity is the mainstay of CPP treatment. Unfortunately, randomized controlled trials have failed to identify an effective stand-alone treatment. Although many patients benefit from skilled manual therapy (PT) as part of a multimodality treatment regimen, access is limited [6]. Additionally, results of physical therapy are less than optimal, with recent studies finding less than 40% pain reduction [7, 8]. Factors including the length of physical therapy treatments and or discomfort may contribute to low patient compliance [9]. The delivery of therapeutic doses of NIR energy to the pelvic muscles may represent a much-needed alternative treatment for those suffering from CPP.

As with medications, the correct dose of NIR energy must be delivered over the correct amount of time to achieve the desired outcome. Numerous investigators have reported optimal irradiance and fluence. Bolton et al. noted that although 810 nm irradiance of 800 mW/cm^2^ at a fluence of 2.4 J/cm^2^ produced greater cell proliferation than irradiance of 400 mW/cm^2^, the higher irradiance lost effect at 7.2 J/cm^2^ [10]. However, at 400 mW/cm^2^ and 7.2 J/cm^2^, proliferation remained significantly increased. More recently, Anders et al. found optimal effects at irradiances between 10 mW/cm^2^ and 50 mW/cm^2^ delivered to a fluence of 200 mJ/cm^2^ [11, 12]. Although an underreporting of irradiance by investigators in the field has hastened the development of a precisely defined dosing regimen, ideal irradiance most likely exists in the range of 10 mW/cm^2^ to 400 mW/cm^2^, and ideal fluence most likely exists in the range of 0.075 to 8.0 J/cm^2^. This dosing must occur at the level of the target tissue and not the overlying skin or mucous membrane. Only a small fraction of the energy applied to the skin or mucous membrane of an organism will reach the target tissue. Hence, the ideal power settings of a laser can only be determined by knowing both the irradiance at the level of the skin and the percentage of the irradiance that will reach the target tissue. Although there may be a consistent pattern of energy decay per millimeter of various tissue types traversed en route to a target tissue, an estimate of such decay is best made for each specific target. The pathway to each unique target tissue represents pathways through unique chromophores. The primary objective of this study is to determine the irradiance needed at the surface of the vaginal mucosa to achieve therapeutic irradiance at the levels of the levator ani muscle and bladder. A secondary endpoint of this study is to determine if this novel transvaginal infrared laser system is capable of delivering such irradiance. As it is critical to incorporate the absorption of water and blood in determining the depth of penetration, a cadaveric study would be insufficient. This study utilizes a live ovine model.

The vaginal structure of sheep is considered similar in structure to humans and provides a sufficiently analogous system for conducting gynecological procedures. Recent histological evaluation of the ovine mucosa and surgical anatomic dissection has validated this analogy [13, 14]. Compared to smaller species, the size of the ovine model also allows for convenient testing of an index device as designed for use in humans. Considering these factors in conjunction with the availability of the ovine model makes this the preferred model for testing a transvaginal treatment device. This research study was conducted in compliance with the requirements of the Animal Welfare Act and amendments and standards in the Guide for the Care and the Use of Laboratory Animals, ILAR, National Academy Press, latest edition as well as GLP, guidelines for nonclinical laboratory studies as described in the Code of Federal Regulations, 21 Part 58, and with any applicable amendments. The study was performed in an Association for the Assessment and Accreditation of Laboratory Animal Care, International (AAALAC) accredited facility and approved by the Institutional Animal Care and Use Committee (IACUC).

## Materials and methods

One healthy, non-pregnant sheep of reproductive maturity was enrolled. Prior to enrollment in the procedure, the animal was housed separately from the opposite sex. Administration of vaginal ointments and medications was avoided for 1 week prior to the procedure. The animal received intravenous sedation followed by the induction of general endotracheal anesthesia. Following induction of anesthesia, the animal was placed in the dorsal lithotomy position. Examination of the vagina demonstrated a normal-appearing mucosa without evidence of trauma or infection. The vaginal length was measured at 11 cm.

**Fig. 1:**
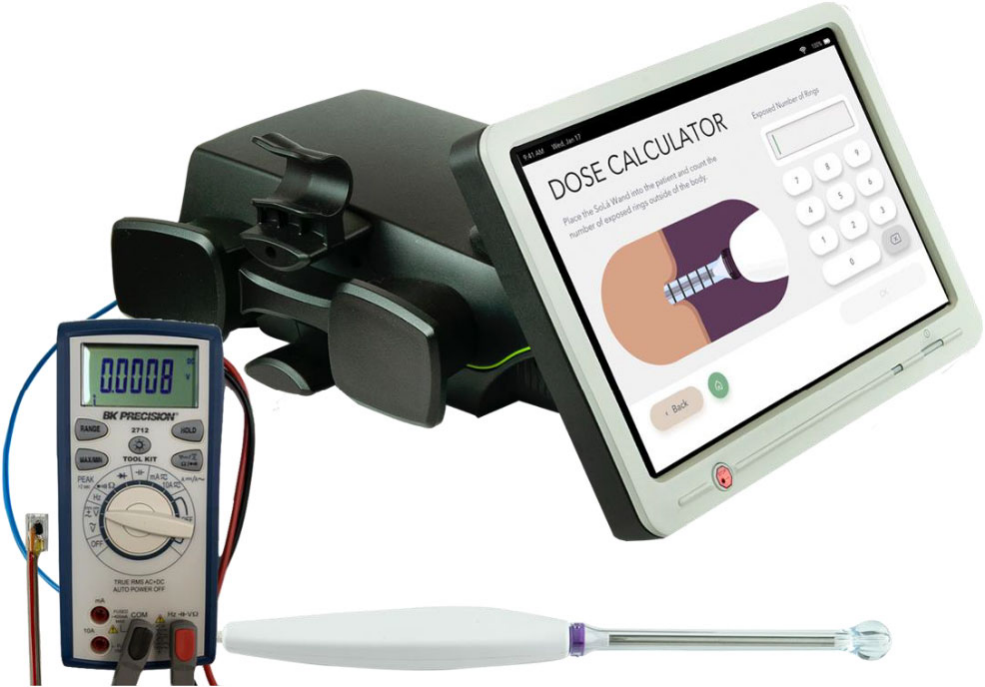
The SoLá Pelvic Therapy Laser System and its transvaginal delivery system. The delivery system consists of a reusable handpiece and proprietary laser fiber that radiates energy perpendicular to the fiber. The distal handpiece is covered by a single-use, sterile, wand with a bulbous tip. Inset is the BK 2712 Precision Multimeter with a custom B&W Tek near-infrared Power sensor. These sensors were implanted in the subject

The laser calibration was confirmed within 40 h of the study and again on the morning of the study. Irradiance measurements were made utilizing the BK Precision 2712 Digital Multimeter and the B&W Tek NIR Power Probe (Fig. 1).

High sensitivity sensors (1 μW/cm^2^) and a low sensitivity sensors  (500 mW/cm^2^) were utilized.

This meter had been calibrated within 7 days of the study by the factory engineers. The Probes were bench tested with the calibrated laser and a control laser (LTS 1500, LiteCure, LLC) for accuracy on the day of the study.

Mucosal surface irradiance was measured by placing the transmitting section of the delivery device (vaginal probe) directly over a Power Probe. Measurements were made at powers of 0.5 and 5.0 W. Next, a 3-cm incision was created in the perineal skin. An American Board of Obstetrician and Gynecologist–certified pelvic reconstructive surgeon performed a transperineal dissection down to the levator ani muscle directly beneath the mid vaginal vault. A 3 × 2 × 1.5 mm Power Probe was placed on the surface of the levator ani muscles. The distance from the perineum to the Power Probe was measured (6 cm). The measurement was used to facilitate vaginal placement of the energy delivery device directly over the Power Probe. A second Power Probe was placed through a tunnel into the belly of the muscle. This probe was placed 1 cm proximal and 1 cm to the right of the more superficial Power Probe. The energy delivery device was placed in the vagina over the superficial (surface) levator ani Power Probe. Irradiance measurements were made at 5.0 W and then 0.5 W. After 5 min, the measurements were repeated in reverse order (0.5 W first). The energy device was next placed over the deeper Power Probe, and irradiance measurements were made at 0.5 W and then 5.0 W. After 5 min, the measurements were repeated in reverse order (5.0 W first). Power Probe was next placed through the urethra and into the bladder. The energy device was next placed in the vagina under the bladder Power Probe. Irradiance measurements were made at 5.0 W and 0.5 W. After 5 min, the measurements were repeated in reverse order (0.5 W first). The bladder was next catheterized of 50 cc of urine. Following euthanasia, the posterior vaginal wall, rectum, and levator ani were excised in-block, and depth measurements were taken (see Table 1).

**Table 1:** Irradiance at target tissue. Irradiance at target tissue provides the laser power in watts (W), a description of each sensor’s position in the subject, the depth of this position from the vaginal mucosa in millimeters, the irradiance at this depth in milliwatts per centimeter squared, and the irradiance at the surface in milliwatts per centimeter squared. The percentage of power lost at depth is described as “irradiance reaching depth”

Power setting (W)	Sensor depth (mm)	Mean irradiance at vaginal mucosa (mW/cm^2^)	Mean irradiance at depth (mW/cm^2^)	Sensor position	Irradiance reaching depth
5	7.5	738	92	Surface of levator ani—beneath mid vaginal vault	12.47%
0.5	7.5	74	9.5*	Surface of levator ani—beneath mid vaginal vault	12.84%*
5	10	738	22	Mid levator ani muscle—beneath mid vaginal vault	2.98%
0.5	10	74	0**	Mid levator ani muscle—beneath mid vaginal vault	0**
5	N/A	738	33	Floating in bladder w/ 50 cc urine	4.47%
0.5	N/A	74	0.02	Floating in bladder w/ 50 cc urine	0.03%

Three measurements were made at each location. Variation was ≤0.5 mW. *Sensor error is ±0.0005 W. **The Power Probe sensors fractured in the tissue tunnel. A measurement was not available

## Results

Approximately 12.5% of the irradiance at the vaginal mucosa reached the surface of the levator ani muscle. Approximately 3% of the initial irradiance reached the center of the levator ani muscle. At a power of 5 W, approximately 4.5% of the irradiance reached the bladder. At 0.5 W, the irradiance reaching the bladder was too low to be detected by the Power Probe (Table 1). Postmortem measurements were made of the harvested posterior pelvic tissues. The distance from the vaginal mucosal surface to the levator ani was 7 mm. The distance to the mid-levator ani muscle belly was 10 mm. The levator ani thickness was 4 mm.

## Discussion

Numerous investigators have reported on the dose-response effect associated with the application of NIR light to living tissue. Although this effect is sometimes referred to as the “biphasic dose-response” effect of light therapy, there is nothing novel about this effect. This effect has been well understood in the pharmaceutical industry for over a century. In the simplest of terms, too small of a dose does nothing. A slightly larger dose may have an effect. A bit more gets the desired effect, and a bit more starts to harm. Take the correct dose over too long of a time interval, and there may be little or no effect, but take that same dose all at once, and you may be harmed. In summary, the correct amount of NIR energy per unit area must be delivered over the correct amount of time. In the dosing of laser energy, these two variables are described by irradiance (power density) and fluence (irradiance × time).

Although both a pilot study and clinical experience have demonstrated the safety and efficacy of this novel transvaginal infrared laser system, this is the first study to document irradiance at the level of target pelvic tissues. This study demonstrates that a novel class IV NIR laser with a novel transvaginal delivery system is capable of delivering therapeutic doses of NIR energy to at least 10 mm below the vaginal mucosa. This depth is sufficient to reach into the human levator ani muscles and bladder, which are found at depths and thicknesses similar to that of the studied animal [15].

The irradiance loss identified in our study is similar to the irradiance loss reported by Anders et al. Ander found that approximately 3% of NIR light transmitted through rabbit epithelium reached the targeted perineal nerve (12 mm deep) [11, 12]. This was nearly identical to our finding at 10 mm. Although each area of the body represents a potential new challenge secondary to varying tissue thickness and chromophores, this variation may represent less of a challenge to class IV NIR lasers. Our study validates the finding of Anders. A low-power laser in the milliwatt range is unlikely to deliver therapeutic dosing to deeper tissues.

Although the loss of irradiance per unit depth appears to be similar across different powers, there was an unexpectedly large drop at 500 mW when measured inside the bladder. Lower irradiances may be more vulnerable to the refraction encountered in urine. Based on the loss of irradiance documented at 5 W, 500 mW should result in irradiance of 2.2 and 3.3 mW/cm^2^ in the levator muscle and bladder. One must remember that the surface irradiance of most class IIIB (milliwatt) lasers is confined to small spot sizes. Small spot sizes impair penetration and will unlikely be able to achieve the 3–4.5% irradiance at the target depths achieved by the studied system herein. Even if such transmission could be achieved, the small spot size would require treatment times that could be prohibitive.

The studied class IV NIR laser is capable of achieving a power of 15 W. Its novel transvaginal delivery system disperses 810 nm and 980 nm light in a 360-degree array. This study demonstrates that this novel system is capable of delivering therapeutic irradiances to and through the levator ani muscle and into the bladder (Table 2). This finding further validates the encouraging therapeutic responses of CPP patients who have been treated with the system since July of 2019.

**Table 2:** Irradiance at surface and inside levator ani muscle by power setting. This table provides irradiance measurements in milliwatts per centimeter squared at the surface of the vaginal mucosa, and the irradiance measurements in milliwatts per centimeter squared reaching the middle portion (in depth) of the levator ani. These measurements are provided at laser power settings of 1, 2, 3, 4, 5, and 6 W

Power (W)	Surface irradiance (mW/cm^2^)	Irradiance inside levator ani* (mW/cm^2^)
1	166	4.95
2	388	11.57
3	522	15.56
4	643	19.17
5	738	22.00
6	998	29.75

*5 W irradiance measured. Other values calculated based on 5 W Power Probe measurement

One weakness of this study is that the data was collected from a live ewe rather than a human. Although the tissues and anatomy are remarkably similar, variations in irradiance transmission may exist. An additional limitation remains the small pool of NIR dose-response data available for the treatment of pelvic muscle spasm and bladder symptoms. Although the data collected during the last year of commercial use of this novel device suggest that the irradiance generated at 5 W is therapeutic and the irradiance generated at .5 W will not be therapeutic in the transvaginal treatment of levator myalgia (myofascial pelvic pain), future clinical studies should gather additional longitudinal data at power settings at, below, and above 5 W.

## Data Availability

Data available within the article or its supplementary materials
